# A Case Report of Decompensated Cirrhosis of Liver with Sideroblastic Anemia

**DOI:** 10.5005/jp-journals-10018-1132

**Published:** 2015-01-06

**Authors:** Mamun-Al Mahtab, Abdur Rahim, Sheikh Mohammad Noor-e-Alam, Faiz Ahmad Khandokar, Ahmed LutfulMubin, Salimur Rahman

**Affiliations:** 1 Department of Hepatology, Bangabandhu Sheikh Mujib Medical University, Shahbag, Dhaka, Bangladesh; 2 Department of Hepatology, Shaheed Suhrawardy Medical College, Sher-e-Bangla Nagar, Dhaka, Bangladesh

**Keywords:** Sideroblastic anemia, Liver cirrhosis, Portal hypertension.

## Abstract

A 35-year-old man was admitted to hospital with severe anemia. Two years back, he had presented with bleeding esophageal varices and subsequently diagnosed as a case of chronic liver disease with portal hypertension. He was then treated with esophageal band ligation. He tested positive for hepatitis B surface antigen with HBV DNA levels of 4 × 10^6^ copies/ml by PCR. His repeat endoscopy showed grade 1 esophageal varices with no evidence of recent or active bleeding. On further investigation, his bone marrow showed dimorphic erythroid hyperplasia with ring sideroblasts and diagnosis of sideroblastic anemia was made. Although rarely, it was suggested that as cirrhosis is a chronic process, it may cause nutritional deficiency, which in turn may lead to the development of anemia.

**How to cite this article:**

Al-Mahtab M, Rahim A, Noor-e-Alam SM, Khandokar FA, LutfulMubin A, Rahman S. A Case Report of Decompensated Cirrhosis of Liver with Sideroblastic Anemia. Euroasian J Hepato-Gastroenterol 2015;5(1):55-56.

## INTRODUCTION

A case of hepatitis B virus (HBV) related decompensated cirrhosis of liver with sideroblastic anemia is reported. The patient is a 35 years old male and a school teacher by profession with low-middle class socioeconomic background. He presented with severe anemia. He had no history of alcohol abuse, neither had family history of such type of anemia. He had no history of taking any possible offending drug on or before his present illness.

On examination, he had stigmata of cirrhosis and mild ascites, which was subsequently confirmed at ultra-sonography. His laboratory investigations showed that his hemoglobin was 6.1 gm/dl, HBsAg and HBeAg positive by ELISA, HBV DNA 4 × 10^6^ copies/ml by PCR, serum bilirubin 26 umol/l, serum ALT 96 U/L, prothrombin time prolonged (control 12 seconds, patient 16.5 seconds) and serum albumin 3 gm/dl. Ultrasonography of hepatobiliary system revealed shrunken liver with coarse echotexture and splenomegaly in addition to mild ascites. The patient had history of hematemesis and melena 2 years back following which esophageal band ligation. Repeat endoscopy of upper gastrointestinal tract showed multiple columns of grade I esophageal varices with no evidence of active, recent or impending bleeding. There was no gastric varix, portal hypertensive gastropathy or gastric antral vascular ectasia. Colonoscopy was also done and revealed normal mucosa and vascular pattern along entire length of the large gut. Stool tested negative for occult blood test.

**Fig. 1:**
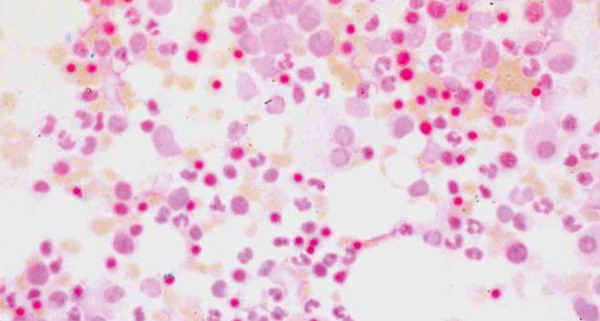
Ring sideroblasts seen in bone marrow of the patient (stained with Pearl’s prussian blue)

Further investigations of the patient revealed normal hemoglobin electrophoresis, negative direct and indirect Coombs’ test, severe anemia with anisochromia, anisocytosis and few microspherocytes in peripheral blood film, hematocrit 0.22, serum ferritin 44.69 ugm/dl and serum vitamin B12 1382pg/ml, serum folate >20 ng/ml. Bone marrow examination was done and showed dimorphic erythroid hyperplasia. Ring sideroblasts were detected in bone marrow when stained with Perls’ Prussian blue, and the diagnosis of sideroblastic anemia was attained (Fig. 1).

## DISCUSSION

Cirrhosis is a chronic liver disease characterized by diffuse fibrosis and nodule formation leading to distortion of normal liver architecture.^1,2^ Clinical evidence of protein-calorie malnutrition and cachexia are seen in a majority of patients with cirrhosis.^3,4^ The sideroblastic anemia is a heterogeneous group of disorders whose two distinctive features are ring sideroblasts in the bone marrow and impaired heme biosynthesis.^5^ Sideroblastic anemia is classified as hereditary or acquired conditions. Etiology, epidemiology, pathophysiology and treatment of these conditions differ vastly. Characteristic feature of sideroblastic anemia is mitochondrial iron deposition.^6^ Other hereditary forms of sideroblastic anemia may have both autosomal dominant and autosomal recessive modes of transmission.^7^ Treatment of sideroblastic anemia begins with ruling out reversible problems including alcohol or other drug toxicity. Treatment is mainly supportive, consisting blood transfusion to maintaining accepted hemoglobin level. A trial of pyridoxine is reasonable. X-linked sideroblastic anemia also responds to pyridoxine.^8^ Folic acid supplementation compensates for possible increased erythropoiesis. Reticulocytosis occurs within 2 weeks in responsive cases, followed by a progressive increase in the hemoglobin level over the next several months. Correction of iron overload improves the response to pyridoxine in some patients with X-linked sideroblastic anemia.^9^ More recently, cytokine therapy with erythropoietin and granulocyte colony-stimulating factor (G-CSF) has been added to the treatment for acquired sideroblastic anemia. Combination therapy appears more effective than single-agent treatment. Transfusion is central in the care of sideroblastic anemia. Symptoms rather than an absolute hemoglobin level or hematocrit should guide transfusion therapy. Even in patients with no meaningful transfusion history, yearly monitoring of the ferritin level and transferring saturation can unveil progressive iron loading. Iron chelation with desferrioxamine is the standard treatment for iron overload, whether transfusional in origin or the result of excessive iron absorption. Occasionally, patients with modest anemia (e.g. hemoglobin 10 g/dl) who are not transfusion dependent will tolerate small-volume phlebotomies to remove iron. In some cases, the anemia improves with removal of excess iron.^10^
